# ICA analysis of fMRI with real-time constraints: an evaluation of fast detection performance as function of algorithms, parameters and *a priori* conditions

**DOI:** 10.3389/fnhum.2013.00019

**Published:** 2013-02-01

**Authors:** Nicola Soldati, Vince D. Calhoun, Lorenzo Bruzzone, Jorge Jovicich

**Affiliations:** ^1^CIMeC, Interdipartimental Center for Mind/Brain Sciences, University of TrentoTrento, Italy; ^2^MIAlab, Department of Electrical and Computer Engineering, The Mind Research Network, University of New MexicoAlbuquerque, NM, USA; ^3^RSlab, Department of Information Engineering and Computer Science, Faculty of Engineering, University of TrentoTrento, Italy; ^4^Department of Cognitive and Education Sciences, University of TrentoTrento, Italy

**Keywords:** independent component analysis, whole-brain fMRI, ill-posed problems, real-time

## Abstract

Independent component analysis (ICA) techniques offer a data-driven possibility to analyze brain functional MRI data in real-time. Typical ICA methods used in functional magnetic resonance imaging (fMRI), however, have been until now mostly developed and optimized for the off-line case in which all data is available. Real-time experiments are ill-posed for ICA in that several constraints are added: limited data, limited analysis time and dynamic changes in the data and computational speed. Previous studies have shown that particular choices of ICA parameters can be used to monitor real-time fMRI (rt-fMRI) brain activation, but it is unknown how other choices would perform. In this rt-fMRI simulation study we investigate and compare the performance of 14 different publicly available ICA algorithms systematically sampling different growing window lengths (WLs), model order (MO) as well as *a priori* conditions (none, spatial or temporal). Performance is evaluated by computing the spatial and temporal correlation to a target component as well as computation time. Four algorithms are identified as best performing (constrained ICA, fastICA, amuse, and evd), with their corresponding parameter choices. Both spatial and temporal priors are found to provide equal or improved performances in similarity to the target compared with their off-line counterpart, with greatly reduced computation costs. This study suggests parameter choices that can be further investigated in a sliding-window approach for a rt-fMRI experiment.

## 1. Introduction

Independent component analysis (ICA) is a data-driven blind source separation (BSS) method widely used in brain functional magnetic resonance imaging (fMRI) data analysis (McKeown et al., 1998; Calhoun and Adali, 2006). The basic idea underlying ICA is to disentangle in a multivariate way all the independent components (ICs) whose combination gives the actual measured signal. The generic procedure is thus to fix an arbitrary number of ICs, i.e., the model order (MO), and let the algorithm exploit a criterion of independence to compute the decomposition that optimizes the criterion given that MO. Several algorithms have been proposed to measure independence of the sources in order to separate them into ICs. The most popular criteria have been based on information theory principles, such as the Infomax algorithm (Bell and Sejnowski, 1995) or higher order statistics (second, third, and fourth order cumulants), such as kurtosis fastICA (Hyvärinen and Oja, 2000). Given the nature of data-driven BSS algorithms which try to deal with and take advantage of an enormous amount of data, ICA found an optimal field of application in the analysis of fMRI data. Its canonical use has been that of analyzing data off-line, that is, once all experimental data has been already acquired. For this paper the use of ICA off-line can be defined as analyzing data in well-posed conditions, as we have usually a great amount of time available for computation and a complete dataset with all the relevant information.

A very different situation arises if ICA is to be considered for dynamic studies such as real-time fMRI (rt-fMRI), in which there is an interest in the dynamic characterization of brain states during the experiment (deCharms, 2008; Weiskopf, 2012). Recently rt-fMRI received a great deal of attention since it makes it possible to perform experiments characterized by novel paradigms (LaConte, 2011; Caria et al., 2012). The most investigated novel paradigm with rt-fMRI is neurofeedback (Shibata et al., 2011; Subramanian et al., 2011). In such experiments subjects receive stimulation that is derived from their ongoing fMRI activity and the task can be to develop mental strategies to regulate the activation. ICA methods could be of interest in such studies for their data-driven nature, particularly when considering experimental designs in which hemodynamic response models will be difficult to use for predicting the brain states under investigation, such as resting state. In a rt-fMRI context ICA will work under ill-posed conditions because the data need to be analyzed under critical time constraints and with a reduced dataset. In addition, since the data changes dynamically whereas the algorithm is usually fixed, the choice of the algorithm can drastically affect computation time and quality of the results. The first implementation of ICA algorithms for rt-fMRI demonstrated successful use of the fastICA algorithm (Esposito et al., 2003). In that work the authors adopted several specific choices for real-time ICA analysis, including a specific ICA algorithm, the choice of a sliding window with a defined temporal window length (WL) and a MO. This study gave two main results. Firstly, it demonstrated in both real and simulated data that the expected task-related activity was equally detected by ICA and by the standard general linear model (GLM) approach. Secondly, ICA was able to detect transient or unexpected neural activity which had not been originally included in the hemodynamic response model. Together these results support the motivation of the evaluation and use of ICA in a rt-fMRI experiments. Real-time ICA has been recently implemented as a plug-in of Turbo Brain Voyager software (Goebel, 2012).

However, there are many possible choices for ICA algorithms, differing mostly in the mathematical criteria used to establish source independence, and it is not obvious which of these algorithms could best characterize neural activity as captured by the BOLD contrast. In addition to which particular algorithm is used, there is also freedom for parameter setting and it is not clear how these might affect the performance of an ICA-based rt-fMRI analysis. Indeed, performance comparisons among different ICA algorithms applied to fMRI data have historically been reported only for the well-posed off-line fMRI case in which the full acquired time-series data was available after the experiment (Esposito et al., 2002; Correa et al., 2005, 2007). Further, from off-line ICA experiments it is known that *a priori* conditions may help the identification of a particular IC most congruent with a predefined target, such as a spatial map (Lin et al., 2010). This *a priori* knowledge can be implemented in different ways depending on the characteristics of the algorithms. It can be as low invasive as a simple tailoring in the nature of the statistical distribution to be extracted, i.e., weighting more super-Gaussian or sub-Gaussian distributions, or as constrained as targeting a specific time course or spatial map. This approach is known as semi-blind decomposition, and its main property is to fuse the positive principles of data-driven algorithms with some kind of *a priori* knowledge on the problem of interest. The introduction of *a priori* knowledge can be done in several ways, e.g., by orienting the decomposition of data into sources with some specific properties. An example of a semi-blind approach is presented in Lin et al. (2010), in which a spatial *a priori* constraint has been introduced in the decomposition algorithm with the aim of extracting the source most congruent with a predefined spatial target. The motivation of considering priors includes reduced computational time (as *a priori* information suggests shortcuts in the decomposition to the algorithm), and improved quality of the sources obtained (given that the results are closer to what is expected). In general not all ICA implementations foresee the possibility of introducing prior knowledge at spatial or temporal level. In this context, and given the noisy data of rt-fMRI experiments from the limited data available for analysis, it is of interest to extend the evaluation of real-time ICA strategies with the consideration of temporal and spatial priors.

In this study we investigated and compared the performance of various ICA algorithms under the ill-posed conditions imposed by rt-fMRI. We used fMRI data of healthy subjects performing a visual-motor task in a framework that simulated a real-time acquisition for each subject separately. Four brain networks were extracted from the full time course of an independent randomly chosen subject not included in further analysis and used as target networks for the performance evaluations: the right and left visual motor networks, the default mode network (DMN), and a noise (NOISE) network associated with physiological noise. In each network we tested 10 out of 14 different publicly available ICA algorithms, and for each algorithm we investigated how the length of the time window (i.e., the number of time points) used for the analysis, the MO (i.e., the number of computed ICs) and the type of *a priori* information (none, spatial or temporal) affected performance. The evaluation of performance was done by considering computation time together with the spatial and temporal correlations of the dynamic ICs with the network reference target. The goal was thus to find, for each network, the ICA implementation that gave the fastest and highest spatial and temporal similarity to the target, but using only a fraction of the time series.

## 2. Materials and methods

### 2.1. fMRI experiment

This simulation study was based on data acquired in a real fMRI experiment (Calhoun et al., 2003). This data set (7 male, 1 female, average age 24 years) has been chosen because it activates a variety of well-known networks (including Default Mode, right visual/motor, and left visual/motor areas) and it has been extensively studied with ICA since part of the dataset is included in the public distribution of the Group ICA fMRI toolbox (GIFT: http://mialab.mrn.org/software/gift/index.html). The dataset is fully described in the original publication and here we outline only the main aspects related to the cognitive tasks, data acquisition and preprocessing.

#### 2.1.1. Cognitive tasks

**Figure 1:**
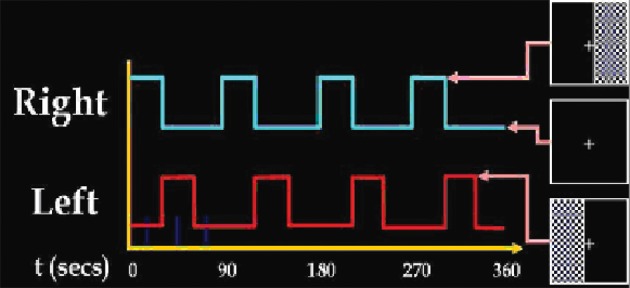
**Summary of the stimulus set-up presented to the subject during experiment data acquisition**.

The visual-motor paradigm contains two identical but spatially offset, periodic, visual stimuli, shifted by 20 s from one another (Figure 1). The visual stimuli were projected via an LCD projector onto a rear-projection screen subtending approximately 25° of visual field, visible via a mirror attached to the MRI head coil. The stimuli consisted of an 8 Hz reversing checker-board pattern presented for 15 s in the right visual hemi-field, followed by 5 s of an asterisk fixation, followed by 15 s of checker-board presented to the left visual hemi-field, followed by 20 s of a central asterisk fixation. The 55 s event set was repeated four times for a total of 220 s. The motor stimuli consisted of participants touching their right thumb to each of their four fingers sequentially, back and forth, at a self-paced rate using the hand on the same side on which the visual stimulus is presented. fMRI data from this paradigm, when analyzed with standard ICA (Calhoun et al., 2003), separated activation network results into two different task-related components, one in left visual and motor cortex, the other in right visual and motor cortex.

#### 2.1.2. Imaging parameters

Scans were acquired by a Philips NT 1.5-Tesla MRI scanner. A sagittal localizer scan was performed first, followed by a T1-weighted anatomic scan [repeat time (TR) = 500 ms, echo time (TE) = 30 ms, field of view = 24 cm, matrix = 256 × 256, slice thickness = 5 mm, and gap = 0.5 mm] consisting of 18 slices through the entire brain including most of the cerebellum. Functional scans were acquired over the same 18 slices consisting of a single-shot, EPI scan (TR = 1 s, TE = 39 ms, field of view = 24 cm, matrix = 64 × 64, slice thickness = 5 mm, gap = 0.5 mm, and flip angle = 90°) obtained consistently over a 3 min, 40 s period for a total of 220 scans. Ten dummy scans were performed at the beginning to allow for longitudinal equilibrium, after which the paradigm was automatically triggered to start by the scanner.

#### 2.1.3. Preprocessing

The data used in this study were previously preprocessed. The fMRI data were first corrected for timing differences between the slices using windowed Fourier interpolation to minimize the dependence upon the reference slice chosen. Next, the data were imported into the statistical parametric mapping software package, SPM99. Data were motion corrected, spatially smoothed with a 6 × 6 × 10 mm Gaussian kernel, and spatially normalized into the standard Talairach space. The data (originally collected at 3.75 × 3.75 × 5 mm) were slightly sub-sampled to 3 × 3 × 5 mm, resulting in 53 × 63 × 28 voxels.

### 2.2. Software and computer for ICA simulations

The entire simulation work was based on an in-house MATLAB (The MathWorks Inc., Natick, Massachusetts) implementation (http://www.mathworks.com/products/matlab) (MATLAB, 2010) that exploits the code available with the GIFT toolbox (GIFT,http://mialab.mrn.org/software/gift). Given the ICA algorithms code present in the toolbox, all the data analysis steps were implemented in an automatic fashion to permit a testing routine to be run on ICA algorithms varying their parameters (i.e., varying the WL, the MO, the *a priori* knowledge, and the subjects). The PC adopted to run the simulations was an Intel(R) Core(TM) i5 CPU M460 @2.53 GHz equipped with 6 GB of RAM and running a Windows 7 64-bit OS.

### 2.3. ICA algorithms

From a total of 14 different ICA algorithms a subset of 10 was considered (see Table 1). Among the algorithms not selected were those based on Infomax criterion, which has been used as reference algorithm, thus it and all the ICA methods based on it (semi-blind infomax, radical ICA, and SDD ICA) were eliminated from the analysis. The algorithms were available from the GIFT toolbox and most of them were discussed in a recent comparative study (Correa et al., 2005). The list included algorithms already used in rt-fMRI experiments, like the fastICA algorithm (Esposito et al., 2003). These algorithms, which are public and were taken as in their original distributions, differ in their data reduction preprocessing steps (e.g., centering, whitening, and dimensionality reduction) and independence criteria for source separation (e.g., minimization of mutual information and maximization of non-Gaussianity) (Cichocki and Amari, 2002).

**Table 1:** **List of tested ICA algorithms and their possibility to accept as parameters arbitrary *a priori* knowledge (both spatial and temporal) and a varying number of ICs**.

**ICA algorithm**	***a priori* knowledge**	**Arbitrary number of ICs**
Infomax	Yes	Yes
FastICA	Yes	Yes
ERICA	No	Yes
SIMBEC	No	Yes
EVD	No	Yes
JADEOPAC	No	No
AMUSE	No	No
SDD ICA	No	No
Semi-blind infomax	Yes	Yes
Constrained ICA	Yes	No
Radical ICA	No	No
COMBI	No	No
ICA-EBM	Yes	Yes
FBSS	Yes	No

*Those algorithms which cannot accept an arbitrary number of ICs extract a number of ICs equal to the time window length. These algorithms references are contained in GIFT toolbox (GIFT: http://mialab.mrn.org/software/gift/index.html)*.

In the following we outline key aspects of the adopted ICA algorithms. A detailed description of each technique is beyond the scope of this study and we refer the reader to the cited works. The selected algorithms cover the major approaches known in the ICA literature for defining independence of sources: information maximization, maximization of non-Gaussianity, joint diagonalization of cross-cumulant matrices and second-order correlation-based methods.

*Infomax* is a stochastic method which uses a non-linear function to maximize the information mapped between input and output of a network. The implementation adopted here was extended infomax, which improves the ability to disentangle sub and super-Gaussian sources using natural gradient descend method (Bell and Sejnowski, 1995; Lee et al., 1999).

*FastICA* is a stochastic method that uses a fixed-point iterative approach to extract maximally non-Gaussian sources. The independence criterion adopted can be higher order statistics or the negentropy of the output (Hyvärinen and Oja, 2000).

*ERICA* (equivariant robust ICA) is an algorithm that minimizes the amount of signal and noise interference on the estimated sources. It is also asymptotically equivariant for sufficient number of samples (Cruces et al., 2000).

*SIMBEC* (simultaneous blind extraction using cumulants) is a deterministic algorithm that exploits natural gradient ascent in a Stiefel manifold with the aim of jointly identify sources using as contrast function higher order cumulants (Amari, 1999; Cruces et al., 2001).

*EVD* (eigen value decomposition) is an algorithm that separates sources exploiting both second-order statistics and higher-order correlation functions. It creates and sums a set of shifted cross variance matrices, after this it applies singular-value decomposition to achieve source separation. The EVD approach is fast and useful when the spectra of the components are different (Georgiev and Cichocki, 2002).

*JADEOPAC* (joint approximate diagonalization of eigenmatrices) is another deterministic algorithm which diagonalizes fourth order cumulant matrices using the Jacobi technique to obtain spatially independent sources (Cardoso and Souloumiac, 1993).

*AMUSE* (algorithm for multiple unknown sources extraction) is a second order method based on the EVD algorithm. The difference is that it applies EVD on a single time-delayed covariance matrix for pre-whitened data. The shift of the cross-variance matrix is chosen here to obtain sources with non-zero autocorrelation of sources at that shift, with auto-correlations as different as possible from each other (Cichocki and Amari, 2002).

*Constrained ICA* is an algorithm that exploits a reference signal to perform ICA. The extracted source is forced to be as close as possible to the reference adopted (Lin et al., 2007, 2010).

*COMBI* is an algorithm which is the result of a combination of two different methods (Combination and Multi-combination of WASOBI and EFICA). SOBI (second order blind identification) was developed with the aim of dealing with sources which could be temporally correlated. It exploits second order statistic to get rid of temporal correlation and maximize the separability of sources (Belouchrani et al., 1993). WASOBI is an asymptotically optimal algorithm for autoregressive sources (Yeredor, 2000), while EFICA is an asymptotically efficient version of the FastICA algorithm (Koldovsky et al., 2006).

*ICA-EBM* (entropy bound minimization) is based on an entropy numerical estimation. The estimated bound of entropy is minimized to find the ICAs. The algorithm adopts a line search procedure initially constraining the demixing matrix to be orthogonal (Li and Adali, 2010b).

*FBSS* (full BSS) is an algorithm that exploits an entropy rate estimator to model second and higher-order correlated sources. This estimator is the adopted to separate sources minimizing their entropy rate (Li and Adali, 2010a).

### 2.4. Use of *a priori* information

As previously mentioned, the exploitation of *a priori* knowledge permits an improvement in the performance of analysis run in ill-posed conditions. However, it is worth noting that the use of *a priori* knowledge can also address another practical challenge of ICA decomposition, which is particularly relevant in ill-posed conditions. In fact a critical choice in ICA algorithms implementation is the ranking or selection of ICs. A practical challenge is to select and track the ICs of interest against the background of non-relevant (or noise) ICs. To address this problem the concept of either spatial (Lin et al., 2010) or temporal (Esposito et al., 2003) *a priori* information has been explored in literature. Other ways to solve the problem of ranking ICs could be represented by exploitation of characteristic expected features of the ICs of interest via a classifier (DeMartino et al., 2007; Soldati et al., 2009).

In the context of rt-fMRI *a priori* information may be available from a localizer scan that elicits aspects of activation that are then to be tracked dynamically in a subsequent experiment. The priors can make the mathematical computation of ICA easier, driving the algorithm initial conditions closer to the basin of attraction of the target IC. In this simulation study the temporal and spatial IC priors were determined from the ICA analysis of the full time series of an independent subject taken from the same group. This *a priori* information was incorporated into the ICA algorithms as an initial estimation of the weighted matrix or as a final constraint of the shape of the target IC. Due to the intrinsic characteristics of the ICA algorithms, only a subset of them allowed us to incorporate spatial and/or temporal *a priori* knowledge in the analysis (see Table 1).

Given the general model of ICA (Calhoun et al., 2001), it is possible to describe an fMRI ICA problem as **Y** = **AX**, where **Y** is the data matrix of dimension equal to the number of time points by the number of voxels; **A** is a mixing matrix of dimension equal to the number of time points by the number of ICs; and **X** is the matrix of the sources of dimension equal to the number of ICs by the number of voxels. If we denote with **W** = **A**^−1^ the weighting matrix (i.e., unmixing matrix), it is then possible to insert *a priori* information in the rows of the matrix **W** directly, if the information is temporal (i.e., a time course). In case the expected or known behavior is spatial (i.e., spatial map) it is possible to construct the **W** matrix as **W** = **Y**pinv**X** where the rows of **X**, i.e., the expected spatial maps of the independent sources are known. In one case [spatially constrained ICA algorithm (Lin et al., 2010)] the *a priori* knowledge is not given as initialization of the weighted matrix but, following the implementation, it is imposed as final target of the decomposition. In this last case instead of starting from a point close to the basin of attraction, the constraint means that the ending point will be close to the basin of attraction. In the context of rt-fMRI *a priori* information may be available from the functional localizer scan that is typically acquired at the beginning of neurofeedback experiments to define the networks that will be of interest to track dynamically.

### 2.5. Parameters analysed in the ICA simulations

The main purpose of this rt-fMRI simulation study was to investigate a number of ICA algorithms to find the one that performed best across subjects using a trade-off of the following parameters:

1. Window length (WL) (i.e., time length of data acquisition)
2. Model order (MO) (i.e., number of ICs)
3. Type of *a priori* information (none, spatial, or temporal)

These choices for these parameters are discussed in more details in the following sections.

### 2.6. Window length and model order

The amount of data that an ICA algorithm uses depends directly on the number of brain volumes available in the growing time window, which in turn defines a limit to the maximum number of ICs that may be computed. As the time WL becomes longer there may be a more accurate representation of the averaged dynamic responses of the brain because more data is available. However, this may come at a cost related to both reducing temporal resolution of the dynamics characterized and increasing the computation time. Conversely, with shorter windows the characterizations may be faster yet less accurate. In this study we focused on a growing window approach because we were interested in finding an optimal WL. For the simulation of each ICA algorithm the WL was varied between 3 and 12 brain volumes (the full time series consisted of 220 brain volumes, and 12 TRs approximated to the hemodynamic delay). For each time WL the number of ICs was varied between 2 (minimum meaningful value of MO in BSS) and the actual WL. Moreover, since for computational reasons the MO must be less than or equal to the WL, the WL minimum value was set to 3. Thus while increasing the WL all possible MOs between 2 and WL were evaluated to find the best performing pair of parameters (WL and MO). Not all the ICA algorithms considered permitted an arbitrary selection of the number of desired ICs. Some of them (jade-opac, amuse, Radical ICA, combi, ICA-ebm, and FBSS) allowed extraction of only the number of ICs that was fixed for each run and was equal to the number of available data points. In our case, this means that for these algorithms the spanned parameter space was represented by a line identified by the points in the space with equal number of ICs and time WL.

### 2.7. Computation template ICs for performance evaluations

Four template ICs were identified on a single subject not included in further analysis by applying the Infomax ICA algorithm with 20 components on the full time series. Infomax is well known to fMRI studies as it has been commonly applied and its performances shown to be stable and reliable (Calhoun et al., 2004). Moreover, when applied on task-related datasets, it furnishes results completely similar to those obtained via application of SPM (Calhoun et al., 2001; Correa et al., 2007). For this reason, although an absolute accuracy as gold standard cannot be defined for ICA results, we opted to use it as a relative reference against which to compare results computed by other algorithms. In addition, to further reduce bias we decided to eliminate from the on-line test analysis Infomax itself and all the other ICA algorithms based on the same criteria (semi-blind infomax, radical ICA, and SDD ICA).

The spatial maps and associated time courses of these networks were later used as reference and as *a priori* knowledge options for the performance evaluation of different ICA implementations, in particular shorter time series to simulate rt-fMRI conditions.

**Figure 2:**
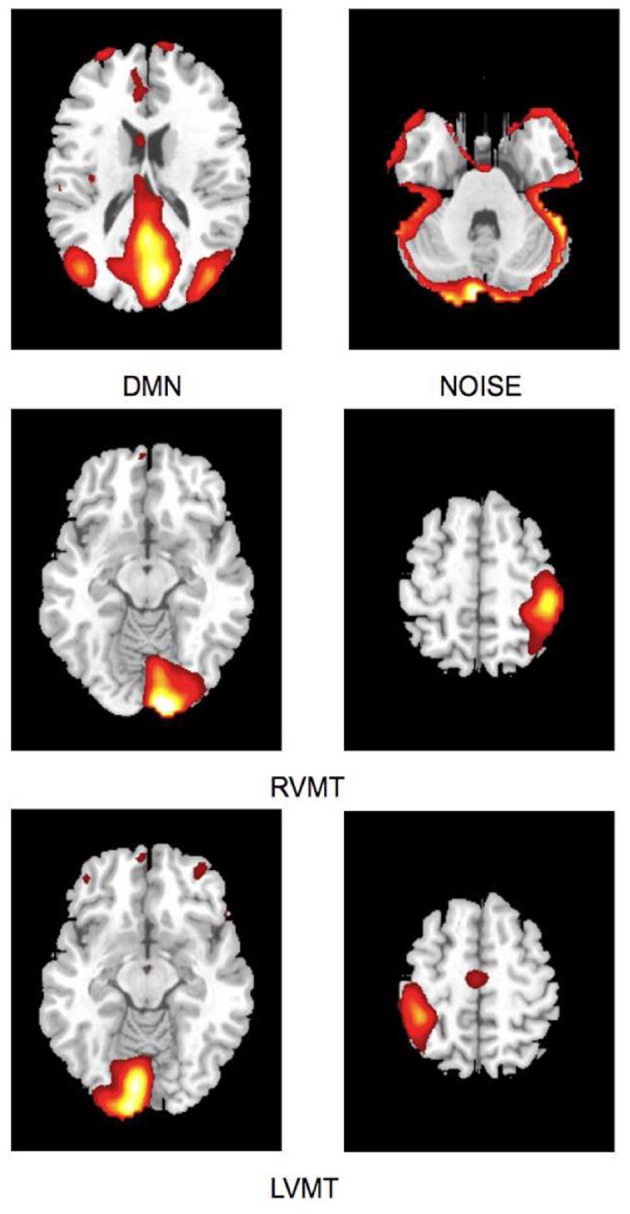
**Spatial maps of ICs considered in the simulation obtained from Group ICA 20 ICs.** For ease of visualization only the relevant slices are reported here. First row depicts default mode network (DMN) and residual motion artifact (Noise). Second and third rows depict the two task-related ICs, right visuo-motor task (RVMT) and left visuo-motor task (LVMT).

The task-related networks were the right visuo-motor task (RVMT) and left visuo-motor task (LVMT), which were selected by visual inspection using as reference the originally published results (Calhoun et al., 2003). In addition, the DMN and a NOISE network were also identified and used as templates for networks typically present in resting state studies (Robinson et al., 2009; Soldati et al., 2009). Figure 2 shows sample spatial representations of the four template networks in a subject.

In our simulation study there could be a potential bias favoring the performance of algorithms that use *a priori* information given that the priors are derived in the same way as the reference templates used for performance estimation: spatial and temporal ICA for the networks of interest using the Infomax ICA algorithm on the full time series. Two considerations were made to reduce this bias. Firstly, a random subject was chosen from the group of 8, the spatial and temporal priors were derived from this subject and used as priors for the other seven subjects. In this way the priors and reference templates are not identical, because the latter ones continue to be calculated for each subject separately. Secondly, the real-time simulation did not use the Infomax algorithm nor other algorithms based on similar principles (semi-blind infomax, radical ICA, and SDD ICA)for performance evaluations.

### 2.8. Evaluation of performance for different ICA implementations

The performance of each ICA algorithm was assessed separately for each subject (7 out of 8) and network (RVMT, LVMT, DMN, and NOISE) by systematically sampling the space of algorithm variables, finding for each variable set the targeted network ICs and comparing them with the corresponding template networks.

The ICA implementations for each subject and network were manipulated through the following variables:

- ICA algorithm: 10 out of 14 algorithms listed in Table 1.
- Prior: all 10 algorithms were tested without priors. A subgroup of four algorithms (fastICA, Constrained ICA, ICA-EBM, and FBSS) allowed the additional implementation of either spatial or temporal priors taken from the template ICs.
- Window length (WL): for each algorithm the WL varied from 3 TRs to 12 TRs in a growing window scheme. The lower limit of 3 TRs was chosen as the minimum time course length for which an ICA can be computed. The upper limit of 12 TRs was chosen because it is approximate to the hemodynamic response.
- Model Order (MO): for each WL the MO was varied between 2 and WL.

**Figure 3:**
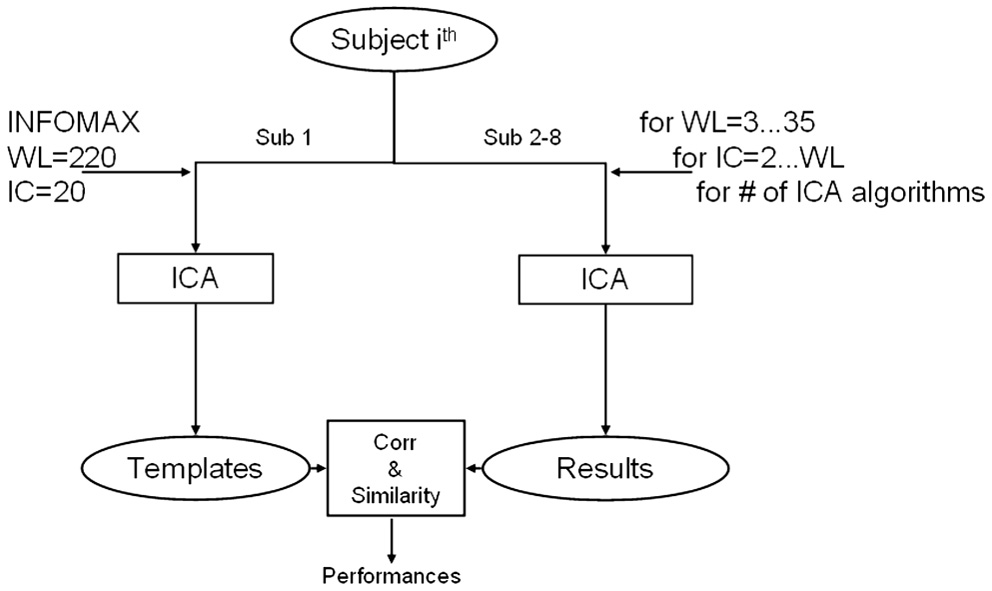
**Diagram of adopted method for ICA algorithm comparison.** For one separated subject data are exploited for creating templates using INFOMAX with model order (ICs) of 20 and window length (WL) equal to the entire available time course. The ICA algorithms are then tested iteratively on all the other subjects for each combination of IC and WL. Results of each computation are compared with templates and evaluated in terms of spatial similarity and temporal correlation.

These parameters were manipulated according to an iterative automatic procedure (Soldati et al., 2010), as schematically shown in Figure 3. This meant that for each subject (a total of 7 out of 8), network (a total of 4), and ICA algorithm (a total of 18: 10 with no priors, 4 with spatial, and 4 with temporal priors), 66 ICA computations were made given that WL spans from 3 to 12 and for each WL, MO spans from 2 to WL. At each iteration the extracted IC results were compared with the templates to estimate the performance of the iteration's parameters.

The performance of each algorithm was characterized from the following three parameters:

1. Spatial similarity with template network: the target network IC was selected automatically by choosing the one with the highest spatial similarity (i.e., spatial overlap) between the ICs extracted and the template IC for the corresponding network. The spatial similarity metric was computed as the absolute value of
  (1) $$\text{Similarity}=\frac{a*b}{\text{norm}(a)*\text{norm}(b)}$$
  where *a* and *b* are the vectors representing the spatial map (reshaped to 1D) of extracted and the template IC of interest, respectively.
2. Temporal correlation with template network: the temporal correlation between the IC extracted and the template IC derived was computed, with its statistical significance (*p* < 0.05).
3. Computation time: the computation time to extract the ICs was recorded.

Considering a fixed subject, brain network and ICA algorithm (with or without prior), the best performing ICA implementation (choice of WL and MO) was considered the one that gave the highest spatial similarity with a significant temporal correlation to the reference network and a computational time below the 12 s threshold.

## 3. Results

The proposed method has been applied to characterize the behavior of different ICA algorithms in ill-posed conditions simulating rt-fMRI manipulating MO, WL, and *a priori* conditions. The goal was to find the implementations that would give the best compromise between computational time and similarity between the detected IC and the reference IC at minimal computation time.

**Figure 4:**
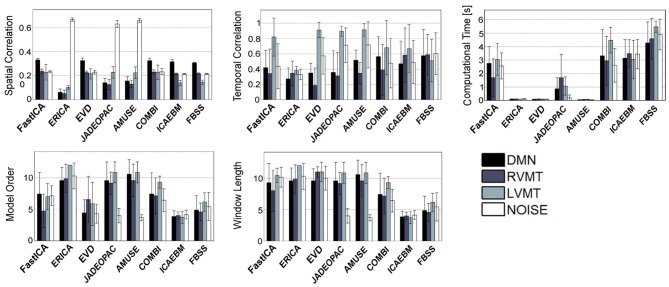
**Results of the best performing runs (mean across subjects) for all available ICA algorithms for a growing length of time window up to 12 TRs and no *a priori* information considered.** For each ICA algorithm the values of similarity (Sim), computational time (CT), temporal correlation (TC), model order (MO), and window length (WL) w.r.t. four reference ICs representing brain activities of interest (Figure 3), are reported for the same optimal condition identified. It is worth noting that here is reported a total of 8 algorithms out of 14 given that Infomax and all those algorithms based on it (semi-blind infomax, radical ICA, and SDD ICA) are excluded from the on-line simulations. Moreover constrained ICA has been excluded since it cannot work without *a priori* knowledge. Finally SIMBEC proved itself to not respect the constraints on computational time, thus it has not been included.

**Figure 5:**
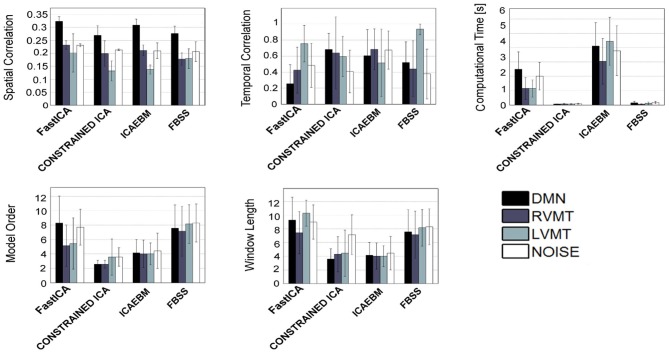
**Similar to Figure 4, but considering only the algorithms which permit the inclusion of spatial *a priori* knowledge**.

**Figure 6:**
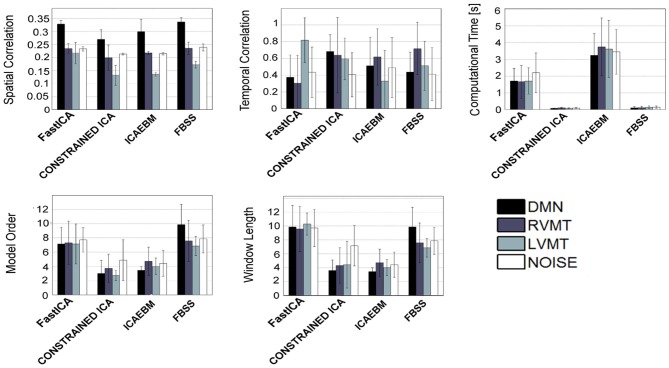
**Similar to Figure 4, but considering only the algorithms which permit the inclusion of temporal *a priori* knowledge**.

The obtained group performance results are reported in Figures 4–6. These figures report the optimal values of the parameters obtained without exploiting *a priori* knowledge (Figure 4), exploiting spatial *a priori* knowledge (Figure 5), or temporal *a priori* knowledge (Figure 6). From the results it can be clearly seen how the selected ICA algorithms differed in performance in these extreme conditions. A trade-off of these results must be obtained to evaluate the winners. In the case of no *a priori* knowledge exploitation (Figure 4) erica, evd, amuse, and partially fastICA seemed to be the more suitable algorithms given their particularly low computational time, with fastICA and evd being the best performing also with respect to spatial and temporal similarity to the reference template.

When considering spatial (Figure 5) and temporal (Figure 6) *a priori* knowledge only 4 of the 10 considered ICA algorithms allowed the evaluation of *a priori* information. Constrained ICA and FBSS were the fastest algorithms, while fastICA, though slower, obtained a slightly higher overall performance in computing similarity metrics. A comparison between Figures 4 and 6 shows the advantages of using prior information with some of the tested algorithms. In particular, for FBSS the computational time improved by a factor of more than two with either spatial or temporal *a priori* information keeping the same performance in terms of spatial and temporal correlation. Also, with the use of *a priori* information the fastest algorithm (constrained ICA, computation time < 0.15 s) was about two orders of magnitude faster than those giving comparable spatial similarities without priors. It is worth noting that the results varied across different monitored networks, i.e., tasks.

## 4. Discussion

The aim of the present study was to evaluate the performance of ICA algorithms in ill-posed conditions, i.e., with a small amount of data availability and constraints on computational time. The issue here was to understand if it is possible to adapt an ICA algorithm to a non-ideal environment, as presented in Esposito et al. (2003). Moreover the analysis was extended to investigate which ICA algorithm was more suitable to this kind of conditions from the perspectives of monitoring a brain activity of interest.

Our goal was to explore the performance in terms of ability to reach the spatial and temporal network characteristics that could be derived from the full dataset in a standard off-line analysis. Thus, we assumed as reference template the optimal results obtained via a single subject ICA with all time-points available, a MO of 20 and using the infomax algorithm, considering stochastic differences not critical. Another intrinsic issue is that the differences in results between off-line and ill-posed conditions can be related not only to computation, but also to the extraction of dynamic behavior with respect to the stationary behavior typically extracted by off-line ICA.

One issue that deserves special consideration is circularity. The use of a validating reference template obtained from the same data used in the simulations did not introduce circularity issues since we are in principle just checking that the same information can be extracted in different ways, with only differences due to noise.

A practical issue to consider is that the high dimensionality of the parameter space results in a high computational load for running simulations spanning the entire multidimensional parameter space. The best performance can be evaluated in a trade-o perspective, since different combinations of parameters can give similar results. The consequence is that performance optimization is heavily connected to the practical application and conditions in which the ICA algorithm is adopted.

Relying on these elements, we performed a direct comparison of different algorithms, defining a cluster of algorithms on the basis of the manipulability of the parameters that they offer (Table 1). In fact the tested ICA algorithms can be divided into three groups: those which accept setting of MO and *a priori* knowledge (i.e., infomax, fastICA, and semi-blind infomax), those which accept neither setting of MO nor *a priori* knowledge (i.e., jade-opac, amuse, radical ICA, and combi), and those which accept only one of the two (i.e., erica, simbec, evd, constrained ICA, ICA-ebm, and FBSS). These constraints are intrinsic to the publicly distributed algorithms. It is beyond the scope of this work to try to change any of the algorithms to eventually make them more flexible. The more flexible algorithms (i.e., those accepting full manipulability of parameters) will, however, not necessarily be better, since the most rigid could be the most adaptable for specific circumstances. Putting everything in a rt-fMRI experiment perspective, it is possible to distinguish the algorithms on the basis of the tasks and conditions they must face. Those algorithms which do not accept any *a priori* knowledge could work very well to define the target networks from the functional localizer step that usually precedes a rt-fMRI acquisition, a step in which *a priori* knowledge may not be necessary or even available. For this use it is possible to permit a higher computational load, since usually the localizer part of an experiment can have more time allocated. The algorithms that tended to be more suitable for this use were evd and amuse, which resulted in particularly fast computation, with evd performing slightly better. The jade-opac and fastICA algorithms also performed well but at the cost of a higher computational time (Figure 5). The results showed that the use of *a priori* knowledge can drastically improve computation time and spatial similarity to a target IC. This suggests that use of priors may be crucial in the dynamic analysis part of the rt-fMRI experiment, where any information from the localizer can be exploited to speed up the process and increase accuracy. From this point of view the flexibility of the ICA algorithm is essential. Thus among the algorithms which accept *a priori* knowledge, constrained ICA provided the optimal solution, followed by fastICA (Figures 5 and 6).

For completeness, it is important to analyze the values of the two parameters growing WL and MO for the previously reported best performing algorithms. In an on-line perspective these values are related to the time needed to elapse before obtaining the first real-time result or step updating. This means that the longer the window and the higher the MO, the more time will pass before the availability of results. This is critical for the on-line computation, since the scale of the resolution in monitoring the brain dynamics will be directly associated to that.

Another observation is related to the type of brain activity monitored (i.e., if it represents a resting state brain activity, a task-related activity or physiological noise). Monitoring ICs with different origins conveys different information. Cross-task variability can be due to the fact that the less the variance of data is explained by the IC, the more difficult it is to extract, especially with a decreased amount of data available. For this reason ICs whose rank is low in a full-data ICA decomposition are critical to identify in the ill-posed conditions. Nonetheless, as the simulations showed, they can still be at least partially captured.

The periodicity of the ICs of interest affects the choice of optimal parameters. The DMN deserves particular considerations due to the low frequency nature of its sources (Damoiseaux et al., 2006). Its identification, despite being easily done by data-driven algorithm, is dramatically harder in ill-posed conditions given that its periodicity is significantly longer than the WL. This results in difficulties in observing its full dynamic. Given these new dimensions (type of brain activity and periodicity) it was possible to see that different algorithms had different effectiveness in adequately identifying brain activity coming from different kinds of sources. It can be seen that the same algorithm could outperform all the others in detecting task-related activity, while suffering in dealing with non-structured noise or, vice versa, as for example it happened in the case of evd and jade-opac, or evd and combi with no *a priori* knowledge. The same reasoning holds for the use of *a priori* knowledge. Even if in this case not all algorithms permitted the introduction of *a priori* knowledge in performing the ICA decomposition, for those which accepted this input the performance varied considering different target sources. Indeed fastICA and constrained ICA alternated best performance, with constrained ICA performing slightly better overall.

Additional ambiguity comes from the stochastic nature of most ICA algorithms, resulting in different runs of ICA delivering slightly different results. This is due to the search procedure of final results optimization, which could result in the algorithm being trapped in a local minima. Another observation can be related to the computational time of ICA decomposition: in general it grows linearly with the increase of the WL, and this can be easily justified by the fact that the more data are to be processed the more time it takes. But as the data become more descriptive of the source to be extracted, the algorithm is able to extract the source more easily, thus reducing the computational time needed, independently of the data length.

One limitation of this study is that the adopted implementations of ICA algorithms are not directly optimized for ill-posed conditions. This opens the door to further development oriented toward their methodological and algorithmic optimization, which would make them more efficient and flexible. Nonetheless, this work demonstrates a methodology for evaluating different ICA implementations for the purpose of finding the ICA algorithms and analysis parameters for the optimal detection of a target brain network under ill-posed conditions. Further experiments are needed to evaluate the performance of ICA implementations on larger datasets and also other networks.

Another element to be taken into account is the relatively small number of subjects adopted in the simulations (8) and reduced number of brain networks studied (visual, motor, and default mode). These constraints result from the use of a dataset whose behavior is well known in the ICA domain and which could confirm the stability and validity of obtained results. Nonetheless, this work demonstrated a methodology for evaluating different ICA implementations for the purpose of finding the ICA algorithms and analysis parameters for the optimal detection of a target brain network under ill-posed conditions. Further experiments are needed to evaluate the performance of ICA implementations on larger datasets, other brain networks and experimental conditions.

The results of this study can be used to evaluate ICA implementations for the dynamic analysis of fMRI data. In particular, in a potential rt-fMRI perspective, the best performing ICA algorithm without the use of *a priori* knowledge can be adopted to analyze the functional localizer data in a data-driven way. In this approach the target ICs to be then followed dynamically in the real-time experiment are defined without considering spatial or temporal constraints. The sources defined by the functional localizer can then be used in different algorithms that include *a priori* spatial, temporal or spatio-temporal knowledge for the dynamic monitoring of target ICs in a rt-fMRI experiment, such as for neurofeedback.

## 5. Conclusion

In this paper we presented an extensive comparison of ICA algorithms under the constraints to have a fast decomposition with a small amount of data available (ill-posed condition). The aim of ICA is to exploit the multivariate nature of data-driven methods to perform a whole-brain analysis. Here we have shown that ICA can satisfactory work in ill-posed conditions with results which are similar and thus acceptable with respect to the off-line implementation. In our comparison we found that several ICA algorithms (evd, amuse, fastICA, and constrained ICA) can be adopted in ill-posed conditions and thus can be exploited for dynamic analysis of fMRI data. The best performing algorithms (evd and constrained ICA) were also shown to be useful in terms of robustness against errors in parameters, and fast in terms of computational time. this opens the door to their exploitation in applications such as rt-fMRI, both as functional localizers and for on-line dynamic analysis. Adoption of these methods would be useful for experimental designs such those known as neurofeedback experiments, although further work is needed to implement a fully real-time ICA method for fMRI data analysis.

### Conflict of interest statement

The authors declare that the research was conducted in the absence of any commercial or financial relationships that could be construed as a potential conflict of interest.
